# Quality of Life and Supportive Care in Multiple Myeloma

**DOI:** 10.4274/Tjh.2012.0192

**Published:** 2013-09-05

**Authors:** Melda Cömert, Ajda Ersoy Güneş, Fahri Şahin, Güray Saydam

**Affiliations:** 1 Ege University School of Medicine, Department of Hematology, İzmir, Turkey

**Keywords:** Multiple myeloma, Quality of life, Supportive care

## Abstract

Multiple myeloma is the second most common haematological malignancy. Novel therapies have led to improvement in survival. Current myeloma management is matching the progress made in improved survival through disease control while optimising quality of life with effective supportive care. Supportive treatment is an essential part of the therapeutic management of myeloma patients because it is directed towards improving the patient’s quality of life and also can improve survival. The aim of this review is to highlight the relationship among life of quality, supportive care, and improvement in survival.

**Conflict of interest:**None declared.

## Abstract (tr)

Multipl miyelom en sık görülen ikinci hematolojik malignitedir. Yeni tedaviler sağkalımda iyileşme sağlamıştır. Günümüzde uygulanan miyelom tedavi yönetimi, hastalığı kontrol altına alarak sağkalımda iyileşme sağlarken etkili destekleyici tedavi ile de yaşam kalitesini artırmaktadır. Destekleyici tedavi, hastaların yaşam kalitesini artırması ve sağkalımı iyileştirmesi nedeni ile miyelom hastalarının tedavi yönetiminin önemli bir parçasıdır. Bu derlemenin amacı yaşam kalitesi, destekleyici tedavi ve sağkalımdaki iyileşme arasındaki ilişkiyi vurgulamaktır.

## INTRODUCTION

Introduction Multiple myeloma (MM) is the second most common haematological malignancy, comprising an estimated 1% of all cancers with an incidence of about 4.5 cases per 100,000 per year [1]. Significant progress in the treatment of MM has been achieved in the past 5 years [2,3,4]. Novel therapies led to improvement in survival, which resulted in an increasing symptom burden due not only to the disease itself, but also to the cumulative effects of treatments [5]. The significant challenge of current myeloma management is matching the progress made in improved survival through disease control while optimising quality of life with effective supportive care from initial diagnosis to end-of-life care [5]. 

It is difficult to define clearly the meaning of the term “quality of life” and it carries different senses for everyone. It involves broad concepts that affect overall life contentment, including good health, adequate housing, employment, personal and family safety, interrelationships, education, and leisure pursuits. Therefore, the life satisfaction most affected by health or illness is defined by the term “health-related quality of life” (HRQoL) [6,7]. Related to HRQoL, we can define supportive care as the treatment given to prevent, control, or relieve complications and side effects and to improve the patients’ and their families’ comfort and quality of life. 

**Quality of Life Questionnaires**

Karnofsky and Burchenal developed a clinical scale to quantify the functional performance of cancer patients in 1949 [8,9] (Table 1). Systematic assessment of HRQoL in cancer patients has received increasing interest over the past 2 decades. Cella et al. defined several advantages to including comprehensive HRQoL surveys in symptom trials in oncology. The most obvious is to test the hypothesis that HRQoL will be improved in addition to the symptom benefits [10]. Nevertheless, assessment of HRQoL has become an important focus of benefit for the treatment of patients with neoplastic diseases [11]. Wisloff reported that measurement of HRQoL before and during treatment contributes important prognostic information [12]. Therefore, it is very important to choose proper assessments and questionnaires. Effective and reliable questionnaires that include generic health status instruments, generic illness instruments, and disease-specific instruments are available for assessment of HRQoL [13]. Among the most widely used cancer questionnaires are the Functional Living Index-Cancer (FLIC), the European Organisation for Research and Treatment of Cancer (EORTC) QLQ-30, a specifically designed instrument for use among patients with MM known as the EORTC QLQ-MY20, and the Functional Assessment of Cancer Therapy (FACT) scale. Moreover, a disease-specific form of the FACT scale was developed through a structured iterative process for MM patients, called FACT-MM. This questionnaire incorporates open-ended questions with classic survey methods, detecting both known and new HRQoL issues for MM patients and the health care providers who treat them [4]. Etto et al. support the use of the EORTC QLQ-C30 as part of routine clinical care in MM patients in developing countries. Their results also suggest that the QLQ-C30 questionnaire for cancer patients seems to be more informative and easier to complete for the patients than the generic questionnaire [5]. The QLQ-C30 incorporates 9 multi-item scales: 5 functional scales (physical, role, cognitive, emotional, and social), 3 symptom scales (fatigue, pain, and nausea and vomiting), a global health and quality-of-life scale, and some single symptom measures. It is available in 16 languages. The EORTC QLQ-MY20 is meant for use among MM patients varying in disease stage and treatment modality (i.e. surgery, chemotherapy, radiotherapy, and hormonal treatment). It should always be complemented by the QLQ-C30. Therefore, it remains difficult to determine the best questionnaire for measurement purposes and patient groups [14]. 

In 2005, the Eastern Cooperative Oncology Group (ECOG) launched a clinical trial (E1A05) to evaluate a new treatment regimen for MM. HRQoL was determined to be a secondary goal for this trial, with the purpose of quantifying treatment-induced reduction of disease-specific symptoms as well as treatment-related symptoms and toxicities. Based on a comprehensive literature search, the authors concluded that no HRQoL instrument existed to adequately capture key MM symptoms and concerns from the patient’s perspective. This led to collaboration among the ECOG Myeloma, Patient Outcomes and Survivorship, and Patient Representative committees to develop a patient-reported outcome (PRO) measure to assess MM symptoms and concerns [4]. The task force identified several types of measures that fall under the PRO umbrella, including HRQoL, functional status, symptom status, overall well-being, satisfaction with care, and treatment adherence. 

**What Affects the Quality of Life? **

Previous studies have demonstrated that the most common physical symptoms indicated by MM patients at presentation are especially skeletal pain and fatigue. Despite significant improvements in the treatment of MM, it remains a chronic incurable disease that is associated with reduced HRQoL due to spontaneous fractures, spinal cord compression, osteolytic lesions, recurrent infections, renal failure, anaemia, mood disorders accompanied by reduced physical functioning, and side effects of different types of treatment used to control this disease [15,16,17,18,19,20,21]. It is an important point that myeloma patients are typically in their sixth to seventh decade of life and have comorbidities. Although the influence of comorbidities in MM patients is yet unsettled, it has been demonstrated to affect progression-free survival and overall survival [22]. This can be considered as an important factor affecting the quality of life. Kleber et al. suggested that assessing the comorbidity status in MM, rather than considering specific age cut-offs alone, may allow better definition of patients’ status, more tolerability of treatment, and more knowledge about the best treatment allocations in upcoming patient cohorts [22]. Concern for the future and loss of labour are other contributing concerns that affect quality of life for younger patients. Blood tests raise anxiety and amplify the emotional effects of the disease. Wagner et al. reported that expert clinicians provided the highest HRQoL relevance ratings for bone pain, bodily pain, difficulty walking, tiring easily, feeling discouraged, interference with activities, and difficulty with self-care as a result of bone pain and fatigue. Quantitative ratings by patients identified sexual function, uncertainty about health, fatigue, weight gain, and emotional concerns, such as worry about new symptoms and difficulty planning for the future, as most relevant to HRQoL [4]. In a prospective population-based study HRQoL and disease-specific complaints of patients with MM up to 10 years after diagnosis were described [23]. The findings of this study showed that patients with MM experience a much lower HRQoL compared to the general population, irrespective of the number of years since diagnosis. Patients with MM reported mean decreases between baseline and 1-year follow-up scores for quality of life (74%), fatigue (50%), nausea and vomiting (71%), pain (59%), and dyspnea (66%). The most bothersome symptoms during the past week were tingling hands/feet (32%), back pain (28%), bone aches/pain (26%), pain in arm/shoulder (19%), and feeling drowsy (18%). Additionally, 37% worried about their future health, 34% thought about their disease, and 21% worried about dying [23]. 

**Treatments and Quality of Life **

Some studies have been conducted about the relationship between myeloma treatments and quality of life. Alegre et al. specified that patients with relapsed or refractory MM treated with long-term lenalidomide reported clinically relevant improvements in certain quality-of-life and symptoms scores regardless of treatment response [24]. Transplant-setting studies have shown that patients’ treatment can have a transient adverse impact on HRQoL [25], but response and improved long-term outcomes are associated with an overall improvement in HRQoL [25,26]. Etto et al. reported that autologous stem cell transplantation (ASCT) improved the quality of life in Brazilian MM patients [5]. Khalafallah et al. reported that dose-reduced tandem ASCT is well tolerated with low toxicity, although it has transient reduction in HRQoL during both transplants. Post-transplant follow-up showed significant improvement in overall HRQoL, reflecting positively on the overall disease outcome [27]. The activity of bortezomib was associated with improved HRQoL in a phase 3 APEX study [28]. Another phase 3 VISTA study showed that there were clinically expressive and statistically significant temporary reductions in HRQoL from baseline in patients receiving bortezomib-melphalan-prednisone (VMP) treatment and relatively lower HRQoL compared with patients treated with melphalan-prednisone (MP), associated with the toxicities arising from the addition of bortezomib to MP [29]. However, the results demonstrated that HRQoL is not compromised in the long term with VMP vs. MP. Moreover, analyses of bortezomib dose intensity indicated better HRQoL in patients receiving lower dose intensity. Additionally, Delforge et al. suggested that clinically and statistically significant improvements in several aspects of HRQoL may occur following response onset in patients achieving an overall response to therapy and particularly complete remission (CR), the rate of which was significantly higher with VMP vs. MP [29]. A recent analysis of the HOVON49 phase 3 trial of MP plus thalidomide (MPT) vs. MP alone in previously untreated elderly MM patients showed that the higher rates of toxicity associated with MPT, despite adversely affecting some HRQoL parameters during treatment, did not negatively affect global health scores vs. MP [29,30]. In a Nordic multicentre trial, 583 previously untreated MM patients were randomised to receive MP or MP + interferon α-2b at a dose of 5 million units subcutaneously, 3 days per week. During the first year of treatment the patients on interferon reported significantly more fever, chills, dry skin, fatigue, pain, nausea/vomiting, and appetite loss than the control patients. There was a moderate reduction of the global quality-of-life score and slight, nonsignificant, reductions of physical, emotional, cognitive, social, and role functioning scores. After the first year there were no statistically significant differences in any toxicity, symptoms, or quality-of-life scores, except for an increased frequency of dizziness in the interferon group [31]. 

**Supportive**

Care The role of the physician in combining life quality and supportive care in MM patients is important because effective supportive treatment results in improved quality of life. Although much of the supportive care can be provided by haematologists, in some patients symptomatic management is achieved through the collaboration of colleagues in the fields of palliative medicine, pain management, clinical oncology, and orthopaedics [6]. A study by Wagner et al. revealed that patients may not discuss more personal aspects of their illness experience (e.g., anxiety, uncertainty, and sexual function) with their physicians. Physicians should educate patients about cognitive dysfunction associated with treatment and should assess them for cognitive decline [4]. 

In addition to chemotherapy, prophylaxis and supportive treatment of bone destruction, pain, anaemia, renal failure, fatigue, infections, hypercalcaemia, and emotional distress are essential parts of the therapeutic management of myeloma patients. The concerted action of supportive therapies can significantly help to maximise the benefits of treatment and to improve the wellbeing of myeloma patients in phases of disease progression as well as during phases of remission. Management of symptoms in patients with myeloma at all stages should follow the principles of evidence-based palliative medicine [6]. Thus, some guidelines were constituted. The aim of these guidelines is to summarise a national consensus of the haematological community and colleagues involved in the supportive care of patients with myeloma [6]. The supportive care definition is sufficiently broad to cover not only symptomatic treatment and palliative care but also the wide range of management options considered to be ‘haematological supportive care’, including anti-infectives, transfusion therapy, anticoagulation, and growth factors [6]. 

**Supportive Care for Bone Disease**

Bone pain, particularly in the spine and chest, is the major symptom in MM, which presents at diagnosis in more than two-thirds of patients. Osteolytic lesions, fractures of long bones, vertebral collapse, and plasmacytomas, which directly affect neural tissues, are the most common causes of bone pain. Later in the course of the disease, pain often arises as a side effect of therapies, e.g., thalidomide or bortezomib neuropathy [32]. Long bone fractures usually require stabilisation by surgical fixation. Radiotherapy may be used as the sole treatment in selected cases, but it should be applied to all lesions prone to fracture. A single 8-10-Gy fraction is recommended [32]. Vertebroplasty by percutaneous injection of low-viscosity liquid bone cement into the vertebral body has been used for pain relief in patients with spine involvement [33]. Kyphoplasty involves the creation of a cavity in the vertebral body and filling it with highly viscous cement, which will result in complete or partial restoration of the collapsed vertebral body. Analgesics, bed rest, and bracing are the other interventions, whose benefits are limited. Some patients may present with or develop instability of their spine or root compression because of primary disease or complications of vertebroplasty, requiring orthopaedic or neurosurgical interventions. Bisphosphonates inhibit bone destruction by blocking the osteoclasts’ recruitment from progenitor cells, suppressing migration, proliferation, and differentiation of osteoclasts and inducing apoptosis of osteoclasts and myeloma cells. Bisphosphonates also inhibit the production of matrix metalloproteinase I and IL-6, which is the most important growth hormone for myeloma cells [33,34,35,36,37]. The efficacy of clodronate, pamidronate, and zoledronate in preventing bone lesions has been investigated in several randomised trials, while for ibandronate limited data from randomised trials are available [32]. Additionally, a network meta-analysis showed superior overall survival with zoledronate compared with etidronate and a placebo. However, there was no difference between zoledronate and other bisphosphonates [38]. Recommended regimens of bisphosphonates are shown in Table 2. Pain improved and quality-of-life and performance statuses were better in patients who received bisphosphonates [33]. Bisphosponate-induced jaw osteonecrosis is an increasingly recognised complication of bisphosphonate therapy that was first described in 2003. Current evidence suggests that the risk is greater for jaw osteonecrosis with zoledronate than with other bisphosphonates. Cumulative dose and duration of treatment are important factors that play a role in this complication [39]. The risk increases in patients who have been taking bisphosphonates for more than 3 years [33]. Thus, bisphosphonates should be discontinued after 2 years of therapy in patients who have achieved CR and/or a plateau phase. For patients whose disease is active, who have not achieved a response, or who have threatening bone disease beyond 2 years, therapy can be tapered to 1 dose every 3 months [32]. 

**Supportive Care for Anaemia **

Anaemia affects more than two-thirds of MM patients [33]. Anaemia is promoted by erythropoietin deficiency, shortened existence of red blood cells, death of immature erythroblasts due to the Fas ligand and TRAIL, decreased responsiveness of the erythron to proliferative signals of erythropoietin, and the myelosuppressive effect of the chemotherapy [32,33]. Anaemia may be managed by red blood cell transfusions in the short-term in a symptomatic patient or by treatment with erythropoiesis-stimulating agents (ESAs) [6]. The improvement with transfusion is transient, and repeated transfusions will be required at intervals of 2-3 weeks. Transfusions have some risks, such as immunological reactions, infections, volume and iron overload, and, in rare cases, even induction of graft-versus-host disease [40]. The high efficacy of erythropoietin in myeloma was documented as early as 1990 in a study on a small number of patients [41]. This pilot study’s findings have been confirmed by several phase 2 and 3 studies. ESAs are the preferred option as they decrease the frequency of transfusions, increase the mean haemoglobin levels, and improve quality of life and performance status [42,43]. It is recommended to start ESAs in patients with haemoglobin levels below 10 g/dL or with haemoglobin levels below 12 g/dL if symptoms due to anaemia are already present [44]. Patients with mild anaemia (Hb 12 g/dL) should be treated because a higher improvement in quality of life occurs when the haemoglobin level increases from 12 to 13 g/dL [33]. However, British guidelines suggest that the haemoglobin concentration should not rise above 120 g/L [45]. Treatment should be initiated with erythropoietin α or β at a dose of 10,000 U 3 times a week or at 30,000 U or 40,000 U once weekly, or with darbepoetin at 150 mg weekly or 500 mg every 3 weeks. The dose can be doubled after 4 weeks in patients with haemoglobin increases of <10 g/dL. ESA treatment should be stopped after 6-8 weeks if there has been no haemoglobin response. ESA doses of <20,000 U/week may be adequate in patients where renal disease is the main cause of the anaemia [6]. American Society of Hematology and American Society of Clinical Oncology guidelines recommend ESAs to be administered at the lowest dose possible and the haemoglobin to be increased to the lowest concentration possible to avoid transfusions [46]. There is an increased risk of thrombotic events and hypertension in patients with cancer who are treated with ESAs [32]. There is a significant improvement in quality of life with a better sense of wellbeing, better exercise capacity, less fatigue, and abrogation of transfusion needs in patients responding to ESAs [47,48,49]. 

**Supportive Care for Infections**

Infections, especially bacterial infections, are frequent complications of MM. Augustson et al. reported that up to 10% of myeloma patients die within 60 days after the diagnosis because of infective causes [50]. Increased predisposition to infections in myeloma is caused by deficits in humoral and cellular immunity, suppression of production of polyclonal immunoglobulins, and use of high-dose steroids in elderly patients or those with poor performance. Active disease is a risk factor for infections. Administration of trimethoprim/sulfamethoxazole at 160 mg/800 mg, twice daily orally during the first 2 months of conventional induction chemotherapy, resulted in significantly decreased frequencies and severities of bacterial infections [51], but routine usage is not recommended because of antibiotic resistance and increased Clostridium difficile infection [6]. Although scientific data on antibiotic prophylaxis are insufficient, several studies suggested that prophylactic antimicrobial therapy should be based on the patient’s risk factors, such as previous history of infections and the type and dose of myeloma therapy [32]. Chapel et al. reported that replacement of intravenous immunoglobulin (IVIG) monthly for 1 year reduced the frequency and severity of infections in plateau-phase patients [52]. A dose of IVIG of 500 mg/kg administered every month for up to 6 months is recommended by guidelines for patients who suffer from recurrent infections and hypogammaglobulinaemia [6,32]. Patients treated with vincristine-adriamycin-dexamethasone (VAD), high-dose dexamethasone, or a bortezomib-based regimens who are at high risk of reactivation or new acquisition of herpetic infections should receive antiviral prophylaxis with oral acyclovir, at 800 mg 4 times daily, or one of the newer antiviral drugs such as famciclovir or valacyclovir [32]. There are also current studies comparing antiviral prophylaxis with acyclovir at 400 mg orally, 3 times daily, and acyclovir at 400 mg once daily in patients treated with bortezomib. There was no statistically significant difference between the 2 groups in terms of herpetic infections [53]. Acyclovir should be given prior to starting to bortezomib treatment and discontinued 4 weeks after the last dose of treatment. Risk factors for infections and the recommended form and dose of prophylaxis are shown in Table 3. Granulocyte colony stimulating factor (G-CSF) may have a role in reducing treatment-associated neutropenia. It is routinely used after autologous and allogeneic transplantations. Addition of G-CSF (5 µg/kg/day) to broad-spectrum antibiotics after high-dose chemotherapy decreases the mortality and morbidity rates, curbs superinfections, and prevents fungal infections [54], but there are also studies that do not support these data. Vaccination against influenza, Streptococcus pneumonia, and Haemophilus influenzae is recommended by guidelines, but the efficacy is not guaranteed [6]. 

**Supportive Care for Peripheral Neuropathy**

Many patients with myeloma have subclinical or even clinical peripheral neuropathy at diagnosis, often due to comorbidities like diabetes mellitus, vitamin B12 deficiency, or carpal tunnel syndrome [6]. This will pose a risk for drug-induced neuropathy with bortezomib and thalidomide. The cause of the neuropathy in many cases of myeloma is not clear, but neurotoxic drugs, amyloidosis, and spinal cord or nerve root compression by plasmacytoma or lytic or extramedullary bone disease are the most common causes [6]. Bortezomib-induced neuropathy (BiPN) is characterised by pain and distal sensory neuropathy with suppression of reflexes, resulting in distal weakness in the lower limbs [55]. Grade 2 neuropathy requires 50% dose reduction of bortezomib and grades 3 and 4 neuropathies require drug discontinuation. Neuropathy grading is shown in Table 4. 

The symptoms of BiPN improve or completely resolve in the majority of patients after a median of 3 months following discontinuation of the drug, while in some patients maximum improvement may take 2 years [56]. Treatment for BiPN is symptomatic relief. Prophylactic treatment is not effective. Thalidomide-induced neuropathy occurs in up to 75% of patients. Daily drug dose, dose intensity, cumulative dose of ≥400 mg, and duration of therapy have been implicated in the pathogenesis. Symptomatic treatment for thalidomide- and bortezomib-induced neuropathy usually comprises gabapentin, pregabalin, or tricyclic antidepressants [57]. Correction of vitamin B12 deficiency and treatment of comorbidities that cause neuropathy are important points for the management of neuropathy. Neuropathic pain scales can be used to define the degree of pain. Guidelines recommend that superficial neuropathic pain should be treated with topical 5% lidocaine plaster and patients with chronic peripheral neuropathic pain should be considered for multimodal analgesic treatment, including an opioid, ion channel blocker, and serotonin-norepinephrine reuptake inhibitors (SNRIs) [6]. 

**Supportive Care for Renal Failure**

Roughly 15%-25% of myeloma patients have a creatinine value of >2 mg/dL at diagnosis. Patients with reversed renal failure have better overall survival than those without improvement [58]. Dehydration, infections, analgesics, hypercalcaemia, and hyperuricaemia increase tubular cast formation. Fluid intake of at least 3 L per day, limitation of analgesic usage, prevention of infections, and oral or intravenous bicarbonate replacement can improve renal functions. The most frequent metabolic complication of MM is hypercalcaemia, predominantly caused by tumour-induced bone resorption by osteoclast-activating factors such as various cytokines and prostaglandins [32]. Symptomatic hypercalcaemia (nausea, vomiting, anorexia, constipation, polydipsia, polyuria, fatigue, confusion, impairment of cognitive functions, coma) requires immediate supportive therapy with 3-6 L/day intravenous saline and high doses of loop diuretics (80-100 mg/day) with frequent evaluations of serum electrolytes [32]. Bisphosphonates can be used for the treatment of hypercalcaemia. 

**Supportive Care for Pain**

**Figure 1:**
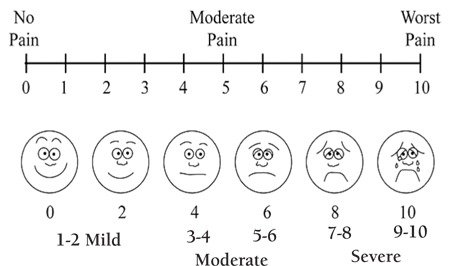
Wong-Baker facial grimace pain scale

Pain is frequently the predominant symptom of myeloma at diagnosis. It is also a common indicator of relapse or progressive disease. Many myeloma patients suffer from pain, particularly in the skeleton [32]. Fractures, osteolytic bone lesions, spinal cord compression, and neuropathy are the most common causes of the pain in myeloma patients. Pain is a subjective experience, and for sufficient treatment, some pain scales must be used (Figure 1). 

Effective analgesia can be achieved by regular administration of oral medication in myeloma patients. The WHO Pain Treatment Ladder (shown in Table 5) has been widely accepted for the treatment of tumour-related pain. 

NSAIDs must be given carefully because of renal toxicity and gastrointestinal effects. COX-2 inhibitors have less gastrointestinal and renal toxicity. Opioids’ long-term effect and tolerance are better, but they are more expensive. The adverse effects of opioids are dryness of mouth, nausea, and emesis. The combination of opioids and NSAIDs is more effective, but also more toxic. Adjuvant medications such as corticosteroids, anti-emetics, neuroleptics, antidepressants, and benzodiazepines should be given as required [32]. 

**Supportive Care for Thromboembolism **

Myeloma and other plasma cell disorders have an association with venous thromboembolism (VTE) [59]. The incidence of VTE is highest during the first 3 to 4 months following the diagnosis [57]. Active disease, infections, previous VTE, and immobilisation are all known risk factors for VTE in myeloma patients. Thalidomide and lenalidomide have been demonstrated to further increase this risk, particularly when combined with steroids or cytotoxic agents [6]. All myeloma patients starting thalidomide or lenalidomide should undergo a risk assessment for VTE. However, the optimal prophylaxis remains controversial. If the patient does not have a risk factor or has only one risk factor (risk factors are shown in Table 6), a standard dose of 325 mg/day or a low dose of 75-80 mg/day of aspirin is recommended, but in the case of 2 or more risk factors, low-molecular-weight heparin (LMWH) at a high-risk prophylactic dose, i.e. enoxaparin at 40 mg, or warfarin (target international normalised ratio [INR]: 2.5) is recommended, unless contraindicated [6]. Aspirin and warfarin showed similar efficacy in reducing thromboembolic events in patients with myeloma treated with thalidomide-based regimens compared with LMWH, but in elderly patients warfarin showed less efficacy than LMWH [60]. The duration of thromboprophylaxis remains unclear, but it is guided by risk factors such as active disease (e.g., for the first 4-6 months of treatment until disease control is achieved) and deescalated or discontinued unless there are ongoing significant risk factors [6]. 

## CONCLUSION

Supportive treatment is an essential part of the therapeutic management of myeloma patients because while directed towards improving the patient’s quality of life, they also have significant effects against the disease and can improve survival [62]. Careful consideration of patients’ and caregivers’ reported symptoms and effective supportive care will result in improved quality of life and improved survival. Some recommendations are given below as clues for supportive treatment from the guidelines. 

**Recommendations [45]**

• A therapeutic trial of ESA should be considered in a patient with persistent symptomatic anaemia (typically haemoglobin concentration of <100 g/L) in whom haematinic deficiency has been excluded. 

• One of darbepoetin, epoetin alfa, or epoetin beta can be chosen. Dose-doubling after 4 weeks in patients with a haemoglobin increase of <10 g/dL can be considered. ESA treatment should be stopped after 6-8 weeks if there has been no haemoglobin response. The haemoglobin concentration should not rise above 12 g/dL. 

• All patients who are due to start thalidomide- or lenalidomide-containing therapy should undergo a risk assessment for VTE. 

• In patients receiving thalidomide or lenalidomide, aspirin (75-325 mg) may be considered as VTE prophylaxis in low-risk patients, but in the case of ≥2 risk factors, LMWH or warfarin (target INR: 2.5) is recommended, unless contraindicated. 

• The duration of thromboprophylaxis remains unclear but is guided by risk factors such as active disease and is deescalated or discontinued unless there are ongoing significant risk factors. 

• Prophylactic immunoglobulin is not routinely recommended but may be useful in patients with severe, recurrent bacterial infections and hypogammaglobulinaemia. 

• Prophylactic acyclovir is recommended for patients receiving VAD, high-dose dexamethasone, or a bortezomib-based regimen who are at high risk of reactivation or new acquisition of herpetic infections and following autologous stem cell transplantation. 

• Pain should be assessed regularly in myeloma patients at all stages of the disease and measured using a 0-10 or a verbal none-mild-moderate-severe scale. 

• Patients who repeatedly score pain as ≥5/10 should be referred to a palliative care or pain team. 

• Effective analgesia can be achieved by regular administration of oral medication. The WHO Pain Treatment Ladder (Table 5) has been widely accepted for the treatment of tumour-related pain. 

• Local radiotherapy is helpful for pain control; a single-fraction dose of 8 Gy is recommended. The use of vertebroplasty or kyphoplasty may be considered in patients with persistent pain. 

• Clinical evidence of a significant (e.g., above NCI grade 2) or progressive peripheral neuropathy at diagnosis should be appropriately investigated to identify treatable causes, and referral to a neurologist should be made so that appropriate neurological investigations can be performed. 

• Patients who develop a significant (e.g., above NCI grade 2) or progressive chemotherapy-induced peripheral neuropathy should be managed with graded dose reduction or drug withdrawal. 

• All patients with chronic peripheral neuropathic pain should be considered for multimodal analgesic treatment including an opioid, ion channel blocker, and SNRI. 

• Dehydration, infections, analgesics, hypercalcaemia, and hyperuricaemia increase tubular cast formation. Fluid intake of at least 3 L per day, limitation of analgesic usage, prevention of infections, and oral or intravenous bicarbonate replacement can improve renal functions. 

## CONFLICT OF INTEREST STATEMENT

The authors of this paper have no conflicts of interest, including specific financial interests, relationships, and/ or affiliations relevant to the subject matter or materials included. 

## Figures and Tables

**Table 1:**
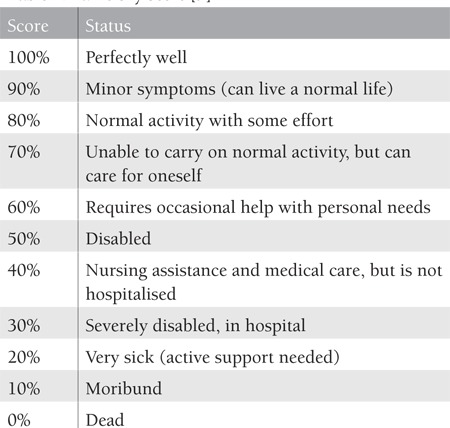
Karnofsky Score [9]

**Table 2:**
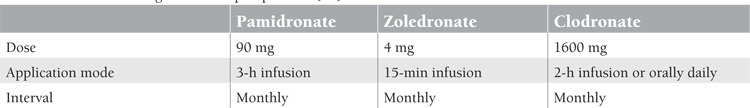
Recommended regimens for bisphosphonates [32]

**Table 3:**
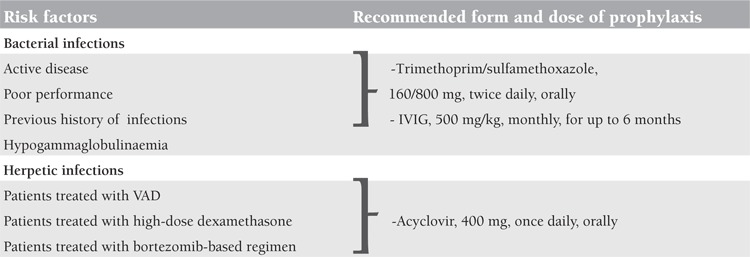
Risk factors for infection and recommended form and dose of prophylaxis in multiple myeloma patients

**Table 4:**
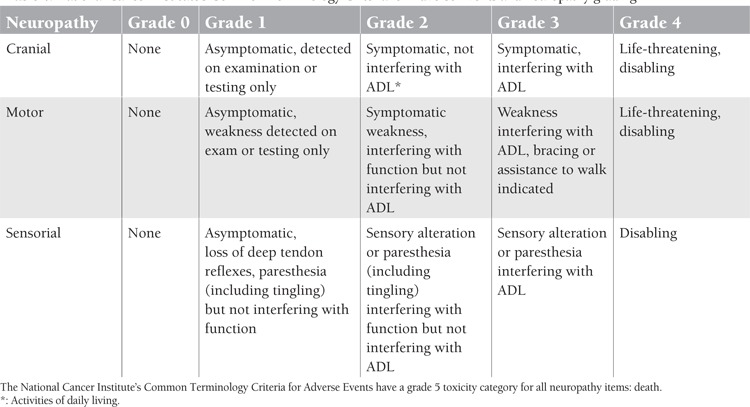
National Cancer Institute’s Common Terminology Criteria for Adverse Events and neuropathy grading

**Table 5:**
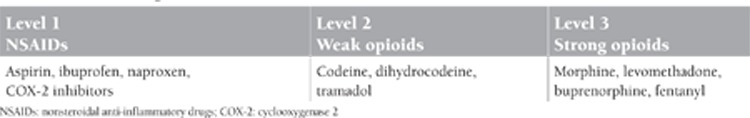
World Health Organization (WHO) Pain Ladder [58]

**Table 6:**
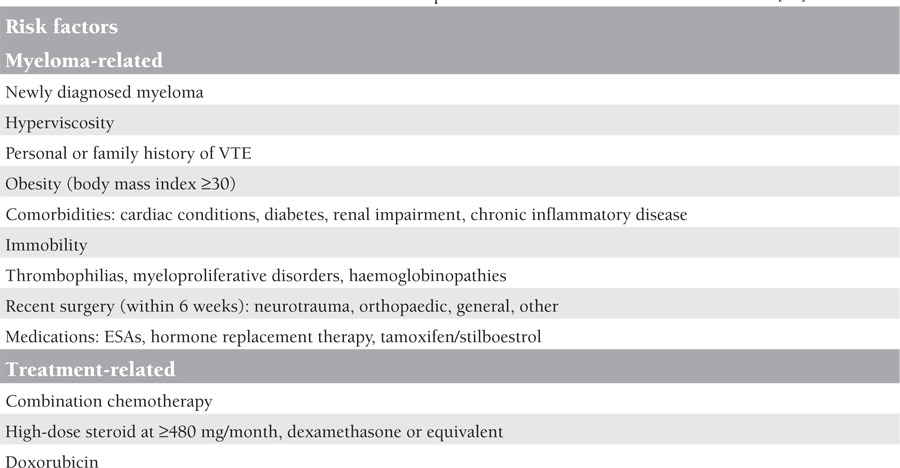
Risk factors of venous thromboembolism for the MM patients treated with lenalidomide or thalidomide [61]
